# LONELINESS AMONG INVOLUNTARILY CHILDLESS OLDER ADULTS: A LIFE COURSE PERSPECTIVE

**DOI:** 10.1093/geroni/igad104.0070

**Published:** 2023-12-21

## Abstract

To this day, having children is seen as the norm, and the consequences in later life when a child wish is not fulfilled are rarely addressed. Therefore, this study investigates the reasons for involuntary childlessness as well as the relationship with potential feelings of loneliness throughout the life course of older adults. Data were collected through individual life story interviews using the McAdams life story interview (N=15) with involuntary childless adults older than 60 living in Belgium. Using a thematic analysis, the results firstly indicate that respondents expressed several reasons for their involuntary childlessness, such as medical reasons, advancing age, career choices and opportunities, partner status and traumas during the life course. Secondly, involuntary childlessness impacts negatively on individual well-being on the one hand, by experiencing feelings of emptiness and deficiency, but on the other hand, having more free time and flexibility during the life course within the social network is perceived as positive. Thirdly, from the moment people realized they would never have children, feelings of loneliness arose. Also, once peers did have (grand)children, or during holiday seasons (e.g., Christmas Day), loneliness feelings became more prevalent compared to earlier in life. However, feelings of loss and emptiness (e.g., missing someone to pass on norms and values to) prevailed over loneliness feelings. The discussion highlights the extent to which older people pick up where they left off and constitute their lives meaningfully at various levels (i.e., at individual level and regarding social relations) when their child wish was never fulfilled.

